# Supplementation of Mother’s Own Milk with Preterm Donor Human Milk: Impact on Protein Intake and Growth in Very Low Birth Weight Infants—A Randomized Controlled Study

**DOI:** 10.3390/nu15030566

**Published:** 2023-01-21

**Authors:** Giannoula Gialeli, Anastasia Kapetanaki, Ourania Panagopoulou, Panagiota Vourna, Athanasios Michos, Christina Kanaka-Gantenbein, George Liosis, Tania Siahanidou

**Affiliations:** 1 Neonatal Intensive Care Unit, “Elena Venizelou” General and Maternal Hospital, 11521 Athens, Greece; 2 First Department of Pediatrics, Medical School, National & Kapodistrian University of Athens, 11527 Athens, Greece

**Keywords:** preterm donor milk, mother’s own milk, growth, VLBW infants, protein, fortification

## Abstract

This randomized study investigates whether feeding very low birth weight (VLBW) infants with mother’s own milk (MOM) supplemented with either preterm (PDM) or term donor milk (TDM), when MOM is insufficient, has a positive impact on infants’ protein intake and growth. A hundred and twenty VLBW infants were randomized into two groups. Group A (43 infants) received MOM supplemented with PDM, whereas Group B (77 infants) was fed with MOM supplemented with TDM, for the first three weeks of life (donor milk period). Breast milk fortifier was added when milk feeds exceeded 50 mL/Kg/day. After the donor milk period, both groups were fed with formula when MOM was not available or the milk bank was unable to provide TDM. Protein intake was higher in Group A than in Group B at initiation of milk fortification (*p* = 0.006), as well as during the 3-week donor milk period (*p* = 0.023) and throughout hospitalization (*p* = 0.014). Moreover, Group A presented higher Δz-score for body weight (*p* = 0.019) and head circumference (*p* = 0.001) from birth to the end of donor milk period, and higher mean body weight at discharge (*p* = 0.047) compared to Group B. In conclusion, when donor milk is required, PDM positively impacts protein intake and growth in VLBW infants (NCT05675397).

## 1. Introduction

Human milk is the best nutritional option for infants, especially those born preterm [1,2,3,4,5,6,7]. Feeding with mother’s own milk (MOM) is considered to be an important factor in increasing the survival and improving outcomes of very low birth weight (VLBW) infants (<1500 g) in the last two decades [8,9,10,11]. However, a considerable number of mothers delivering preterm struggle to provide adequate milk the first days after delivery to meet their infants’ requirements. When MOM is not sufficient, pasteurized donor milk (DM) is the best alternative according to current recommendations [7,12,13,14].

Nowadays, many maternity hospitals have organized milk banks in order to cater the needs of premature infants. Donor milk is primarily derived from mothers of term-born infants for the first six months of lactation [15]. This term milk presents significant differences compared to preterm human milk which has higher protein concentration and more caloric energy [16]. According to Bauer et al., protein content in extremely preterm human milk versus term human milk differs by 0.73 g/dl and provides almost 10 kcal/dl more energy [17].

According to ESPGHAN guidelines, VLBW infants have high needs of protein intake soon after birth [18]. For the first critical week of life, Stephens et al. have demonstrated that an increase in protein intake by 1 g/kg/day is associated with better neurodevelopmental outcome [19]. After this short period, preterm MOM needs fortification in order to meet the nutrient requirements of VLBW infants whose needs are dynamic and related to postnatal age, severity of illness, and need for catch-up growth [20,21,22]. In most neonatal intensive care units (NICUs), fortification is initiated when the fed milk volume reaches 50–100 mL/kg/day [23,24]. However, for VLBW infants, this delay may result in significant protein deficit during the first weeks of life. This is a primary concern in infants fed with term donor milk given the fact that protein amounts in human milk tend to decrease with postnatal age [25].

We hypothesized that feeding VLBW infants with preterm donor milk (PDM) in combination with MOM may positively influence the protein intake and, consequently, the infants’ growth. The aim of the current study was to assess whether MOM supplementation with PDM has any beneficial effects on energy and protein intake and growth in VLBW infants.

## 2. Materials and Methods

### 2.1. Study Design and Participants

This is a randomized, controlled, double-blind study conducted in a level III NICU, at “Elena Venizelou” General and Maternal Hospital in Athens, Greece. VLBW infants with birth weight <1500 g and born to mothers who had agreed to feed their babies with donor milk for the first three weeks of life (donor milk period) if their own milk quantity was insufficient were enrolled in the study from April 2017 to August 2021. Infants were excluded if they had congenital anomalies, chromosomal disorders or metabolic diseases. Also, any infant fed with formula at any point during the donor milk period was not eligible to participate in the study.

The Ethics Committee of “Elena Venizelou” General and Maternal Hospital approved the investigation and each infant’s parent provided written informed consent before participating in the study.

### 2.2. Eligible Donor Mothers

Mothers who gave birth at <35 weeks of gestation could become donors of preterm milk (PDM) until they reach 34^+6^ weeks of corrected gestational age and only during their first four weeks of lactation. Also, the Milk Bank of “Elena Venizelou” hospital provided term donor milk (TDM) from eligible mothers who expressed breast milk for a period up to six months post-delivery. All eligible donor mothers were serologically screened for infectious diseases including hepatitis B and C, Human Immunodeficiency virus, Cytomegalovirus, and Syphilis [26]

### 2.3. Collection and Storage of Donor Human Milk

DM was collected in a special container and was processed by the “Elena Venizelou” Milk Bank using the Holder Pasteurization method (human milk was heated to 62.5 °C for 30 min) and quickly cooled down to 25 °C within 10 min in an ice water bath; it was then stored in a refrigerator at a temperature of 0–4 °C for one to two days or frozen at −20 °C for three to six months. Single donor expressed human milk was collected and pasteurized separately.

### 2.4. Feeding Protocol and Analysis of Human Milk

Following enrollment, infants were randomly assigned using a sequentially numbered sealed opaque envelope system with a ratio 1:2 to be fed with MOM supplemented, if required, either with PDM (Group A) or with TDM (Group B). If MOM was completely unavailable, the infant was fed with pasteurized PDM or TDM following randomization. Neither the attending physicians nor the nurses were aware of the type of donor milk. Only the principal investigator (not involved in the care of enrolled infants) knew the type of donor milk provided.

Pasteurized PDM or TDM, as supplementary to MOM or for exclusive feeding, was provided to the study population for a three-week period after birth (defined as donor milk period) during which time the milk was analyzed regularly for protein and calorie content. After the donor milk period and until discharge, increasing demands were met with formula supplementation if MOM was not available or the milk bank was unable to provide TDM.

Parenteral nutrition was started immediately after birth for all infants. Feeding of unfortified ΜOΜ or pasteurized DM from the hospital’s milk bank was initiated within the first three days of life. Powder breast milk fortifier (BMF) was added to human milk when milk intake reached 50–100 mL/kg/day. In all cases, a standard fortification with BMF (Nutricia Human Milk Fortifier) was applied (two sachets BMF per 100 mL of milk) according to the manufacturer’s instructions. Each sachet of fortifier (2.2 g) provided 0.6 g of protein in the form of extensively hydrolyzed bovine whey protein and 8 kcal of energy. The fortifier was introduced at half dose (1 sachet per 100 mL) for two to three days and then increased to full dose (2 sachets per 100 mL).

Since sampling and pooling of milk samples after a full breast expression at each feed across a 24-hour period is considered the “gold standard” for human milk collection [16,27], MOM was collected throughout the last 24 h. Frozen DM (PDM or TDM) was thawed before it was administered to neonates within 24 h. A random representative sample of the human milk pool (MOM or DM) was analyzed, before fortification, once a week using a MIRIS Human Milk Analyzer (HMA™, Miris AB, Uppsala, Sweden). The Miris HMA is based on semi-solid mid-infrared (MIR) transmission spectroscopy. A calibration check was performed prior to analysis using the calibration solution provided by the supplier. For the analyzer, a 2 mL sample of milk was required, which was heated at 40 °C in a thermostatic bath before undergoing analysis. In this study, Miris HMA was used to record the amount of protein and caloric energy content of milk samples.

### 2.5. Data Collection

The following parameters were recorded in all infants during hospitalization:

- CRIB II score (Clinical Risk Index for Babies), a validated measure of initial mortality risk and illness severity within the first hour of admission [28].
- Anthropometric characteristics (body weight and length, head circumference) at birth, at the end of donor milk period and at discharge. The anthropometric characteristics were recorded as both absolute values and z-scores; z-scores were documented according to the revised Fenton growth charts using the PediTools [29]. Small for gestational age (SGA) status was defined as birthweight z-score less than −2 SD from the mean. The difference (Δz-score) of z-score for body weight, body length and head circumference from birth to the end of donor milk period, as well as from birth to discharge, was calculated.
- Total protein and energy intake through parenteral nutrition, MOM, DM and fortification.
- Neonatal/Infant Morbidity. In particular:
  - Culture-positive sepsis (clinical signs of infection plus positive blood, urine or cerebrospinal fluid culture).
  - Necrotizing Enterocolitis (NEC) if fulfilling criteria compatible with Bell’s stage 2 or higher.
  - Bronchopulmonary dysplasia (BPD) defined as need for supplemental oxygen for more than 28 days.
  - Retinopathy of prematurity (ROP)—if fulfilling criteria compatible with Stage 3 according to international classification of retinopathy of prematurity (2005) [30].
  - Intraventricular hemorrhage (IVH)—any degree.
  - Patent ductus arteriosus (PDA) (presence of clinical signs plus ductal left to right shunt in echocardiography).

### 2.6. Statistical Analysis

Quantitative variables were expressed as mean values (standard deviation; SD) or as median (interquartile range; IQR), while qualitative variables were expressed as absolute and relative frequencies. For comparison of proportions, chi-square and Fisher’s exact tests were used. Independent samples Student’s t-tests or Mann-Whitney U tests were used, as appropriate, for comparisons between Group A and Group B. Repeated measurements analysis of variance (ANOVA) was applied to evaluate changes in total protein intake between groups over the donor milk period. In order to evaluate the change in pasteurized donor milk quantity received by the study population over the donor milk period, linear mixed regression was applied and regression coefficients (β) with standard errors (SE) were computed. Sensitivity analysis was also run after excluding infants born to mothers with multiple pregnancies. All reported *p* values are two-tailed. Statistical significance was set at *p* < 0.05 and analyses were conducted using the SPSS statistical software (version 22.0).

## 3. Results

**Figure 1:**
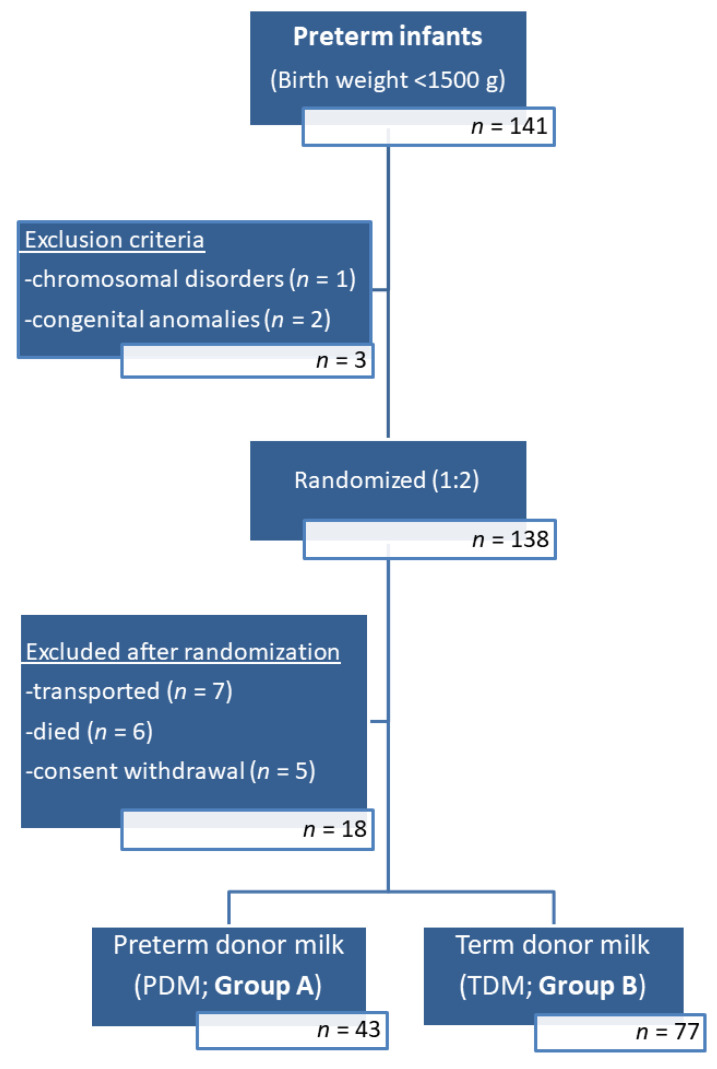
Flow chart of the study population.

A hundred and thirty-eight VLBW infants were enrolled in the study. Of them, 18 infants were excluded after randomization due to transportation, death before discharge or parental decision to withdraw the consent for their child participation. Finally, 120 infants allocated to Group A (*n* = 43) or Group B (*n* = 77) were studied (Figure 1).

Maternal age, gestational age, multiple pregnancies, infants’ gender, anthropometric characteristics at birth, SGA status, CRIB II score and length of hospitalization did not differ significantly between Group A and Group B (Table 1).

PDM and TDM for feeding were derived from 26 and 43 donor mothers, respectively. As expected, gestational age was significantly lower in mothers who provided PDM compared to mothers who provided TDM (*p* < 0.001). No difference was recorded in maternal age (*p* = 0.130), parity (*p* = 0.100) and multiple gestation (*p* = 0.138) between preterm and term donor mothers (Table 2).

The proportion of pasteurized DM (PDM or TDM) diminished significantly over time (β = −3.56; SE = 0.47; *p* < 0.001). However, the degree of change did not differ significantly between the two groups (β = −0.35; SE = 0.98; *p* = 0.719). Moreover, following the donor milk period, no difference was recorded in the proportion of formula supplementation until discharge between groups [mean (SD): 27.9 (24.4)% in Group A vs. 21.7 (25.2)% in Group B; *p* = 0.123).

### 3.1. Protein and Calorie Intake

Mean protein intake during the donor milk period was significantly higher in Group A than in Group B (*p* = 0.023). When protein intake was assessed at each week separately, a significant difference between groups was observed at week 3 after birth (*p* = 0.01).

Protein intake was also significantly higher in Group A than in Group B at initiation of breast milk fortification (*p* = 0.006) and throughout hospitalization (*p* = 0.014) (Table 3).

Calorie intake during the donor milk period [median (IQR): 115.4 (105.0–129.7) kcal/kg/day in Group A vs. 114.0 (101.3–133.8) kcal/Kg/day in Group B] and during the entire hospitalization [107.0 (96.0–116.5) kcal/kg/day in Group A vs. 102.3 (92.6–111.2) kcal/kg/day in Group B] did not differ significantly between the two groups (*p* = 0.782 and *p* = 0.164, respectively).

### 3.2. Growth

**Figure 2:**
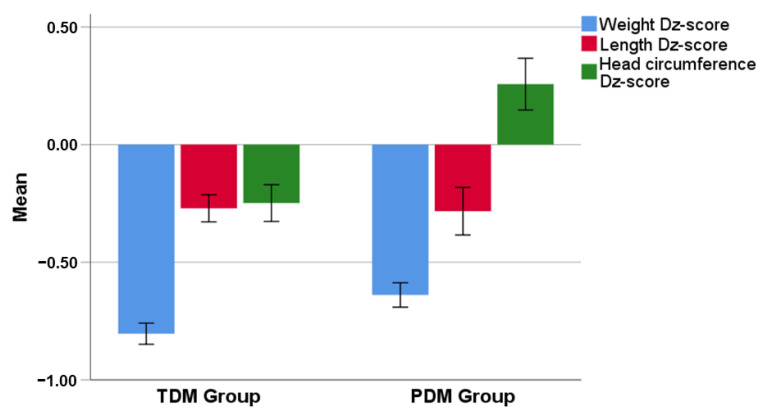
Δz-scores for body weight (*p* = 0.019), body length (*p* = 0.720) and head circumference (*p* = 0.001) from birth to the end of intervention (donor milk period) between preterm (PDM) and term donor milk (TDM) Group.

At the end of donor milk period, no difference was recorded between Group A and Group B in body weight z-score [−1.12 (0.87) vs. −1.28 (0.84); *p* = 0.309), body length z-score [−0.41 (1.00) vs. −0.48 (1.06); *p* = 0.705) or head circumference z-score [−0.13 (0.95) vs. −0.38 (1.14); *p* = 0.215). However, Δz-score in body weight and head circumference from birth to the end of donor milk period differed significantly between Group A [−0.63 (0.34) and 0.26 (0.72), respectively] and Group B [−0.81 (0.39); *p* = 0.019 and −0.22 (0.72); *p* = 0.001, respectively]. No difference was found in Δz-score for body length between groups [−0.28 (0.66) vs. −0.25 (0.56); *p* = 0.720) (Figure 2).

At discharge, body weight was significantly higher in Group A [mean (SD): 2560.7 (423.4) g] than in Group B [2411.8 (370.5) g; *p* = 0.047] (Figure S1). However, body weight z-score [−1.61 (1.02) in Group A vs. −1.70 (0.94) in Group B] and Δz-score from birth to discharge [−1.13 (0.68) in Group A vs. −1.23 (0.86) in Group B] did not differ significantly between groups (*p* = 0.627 and *p* = 0.542, respectively).

Body length z-score [−0.92 (1.39) vs. −0.97 (1.11); *p* = 0.814) and Δz-score from birth to discharge [−0.79 (1.02) vs. −0.73 (0.73); *p* = 0.708), as well as head circumference z-score [−0.46 (0.98) vs. −0.55 (0.97); *p* = 0.643) and Δz-score [−0.08 (1.24) vs. −0.39 (1.11); *p* = 0.169) did not differ significantly between the two groups.

In the sensitivity analysis, after excluding infants from multiple pregnancies, the results of the study did not change.

### 3.3. Clinical Characteristics and Morbidity during Hospitalization

No significant difference was found between groups in time to regain birth weight [median (IQR): 8.0 (7.0–13.0) days in Group A vs. 10.0 (8.0–14.0) days in Group B; *p* = 0.09] or in time to full enteral feeds of at least 150 mL/Kg/day [12.0 (9.0–18.0) days in Group A vs. 14.0 (10.0–21.0) days in Group B; *p* = 0.245].

Morbidity during hospitalization did not differ significantly between groups (Table 4).

## 4. Discussion

Optimal growth of VLBW infants is a primary goal in NICU but not easy to achieve. Growth failure was observed in 80% of VLBW infants in studies conducted under the Neonatal Research Network between 2008 and 2010 [31]. In 2013, among infants cared for in hospitals affiliated with the Vermont Oxford Network, 50.3% demonstrated growth failure at the time of hospital discharge [32]. Similarly, according to data obtained from the Korean Neonatal Network database from 2013 to 2014, the overall incidence of postnatal growth failure in VLBW infants was 45.5% [33]. Providing adequate nutrition is of paramount importance since growth failure has been associated with neurodevelopmental impairment. Especially protein intake has been strongly linked to the accomplishment of optimal growth and development in VLBW infants [34,35]. Stephens et al. have showed that an increase in protein intake by 1 g/kg per day during the first week of life was independently associated with more than an 8-point increase in Mental Developmental Index (MDI) at 18 months of age [19]. Growth failure occurs when nutrient intake is inadequate over a period of time. The exact timing and duration of this inadequacy has yet to be determined exactly in the preterm population. According to ESPGHAN guidelines [18] based on the protein needs and nitrogen utilization, the protein intake should be at least 3 g/kg/day for preterm infants up to a weight of approximately 1800 g. Unfortunately, in most studies the exact composition of human milk is unknown and clinicians estimate the nutrient content of each mother’s milk according to specified references [16].

In this study, the composition of milk was accurately measured using a human milk analyzer (Miris HMA); the analyzer was used to capture the exact composition of MOM and DM at set time intervals. Miris HMA was not used as a tool for individualized fortification since breast milk samples in both groups were similarly fortified per manufacturer guidelines and regardless of measured protein concentration in order to fulfill the goals of the study. It is well known that the protein concentration of milk from mothers delivering preterm is initially higher than that from mothers delivering at term [36] but decreases after the first weeks of lactation [17]. This is the reason why, in this study, preterm donor milk was used only if expressed within the first four weeks after childbirth [37]. Maternal age, parity and multiple gestations that might have also impacted the breast milk nutrients composition [38,39] did not differ significantly between the two study groups.

The results of this study indicate that protein intake was greater in VLBW infants when MOM was supplemented with preterm rather than term donor milk. These infants fulfilled during the donor milk period the minimum protein requirements according to both ESPGHAN guidelines (3 g/kg/day) [18] and EMBA working group recommendation (3.5–4.5 g/kg/day) [24]. It is worth noting that on the day of initiation of human milk fortification, protein intake was significantly higher in Group A compared to Group B (3.57 g/kg/day vs. 2.92 g/kg/day, *p* = 0.006) which indicates the higher provision of protein by preterm donor milk *per se*. Although both groups achieved over 3 g/kg/day of protein intake during the first vulnerable week of life, as well as throughout the whole donor milk period, only Group A surpassed the amount of 3.5 g/kg/day which is in line with the most recent recommendations [24,40]. However, more studies are needed to determine the exact protein requirement for this population and whether it should be different in the extrauterine environment compared to the intrauterine one.

The above results demonstrate that feeding VLBW infants with PDM, when MOM is not sufficient, and starting fortification when the quantity of enteral feeds reaches 50–100 mL/kg/day, protein intake surpasses the amount of 3.5 g/kg/day as ESPGHAN (2022) recommends [40]. The precise time to commence fortifiers is not known. Few data exist whether early (<40 mL/kg/day) versus delayed (>75 mL/kg/day) initiation of fortification may be beneficial [41]. Current recommendations of ESPGHAN propose starting a fortifier when enteral intakes reach 40–100 mL/kg/day [40]. An individualized fortification strategy (i.e., adjustable or targeted) was also proposed [40].

Overall, there are great discrepancies in literature regarding the effect of early nutrition on anthropometric parameters of VLBW infants, especially when pasteurized donor milk is used. It is important to note in previous studies whether the results were documented before or after the practice of human milk fortification was initiated. Historically, before the era of human milk fortification, studies showed that infants fed with MOM supplemented with pasteurized DM exhibited growth retardation based on anthropometric measurements of body weight, length and head circumference over a period of time [42]. In contrast, studies conducted after the implementation of human milk fortification have shown that VLBW infants fed with MOM supplemented with donor milk have similar or even faster growth rates than the ones fed with MOM supplemented with preterm formula [43]. Ginovart et al. studied two groups of VLBW infants. The first group included 72 infants born prior to the initiation of human milk fortification and fed exclusively with formula. The second group included 114 infants born after the implementation of fortification of MOM and DM. The conclusion of that study was that formula may not be appropriate for initiation of feeding in preterm infants [44]. On the contrary, in a 2017 study by Madore et al. [45], very preterm infants fed MOM supplemented with donor milk had lower weight gain during the first month of life than those fed only MOM or MOM supplemented with preterm formula. Thus, further studies on this issue are needed.

In 2014, Dritsakou et al. [15] compared two groups of VLBW infants; one fed with MOM supplemented with term donor milk until discharge and the other fed exclusively with donor milk for the first three weeks of life followed by formula, if necessary, until discharge. Human milk was fortified in both groups. The first group showed greater head circumference and body length, whereas there was no difference in body weight between the two groups [15]. In the present study, donor milk is further categorized by mothers’ length of gestation at birth (term versus preterm). No difference was found in infants’ body length; however, Δz-score of body weight and head circumference from birth to the end of intervention differed significantly between groups. Moreover, body weight at discharge was by 6.2% higher in Group A than in Group B; this fact indicates that the nutrient composition of preterm versus term donor milk has a positive impact on infants’ growth. It is also worth noting that Group A infants reached full enteral feeding two days earlier than Group B which is very important since it limits the use of indwelling catheters and the associated risk of infection [31].

The novelty in the design of this study was the comparison of PDM with TDM. To our knowledge, this is the first study that investigates the potential benefits of preterm donor milk on protein intake and growth in preterm infants. Only Fang et al. [46] evaluated recently, in 2021, whether preterm donor milk might have any beneficial effects in VLBW infants. However, in that study, preterm donor milk was compared to formula, not to term donor milk. Moreover, preterm donor milk was firstly expressed from mothers at 3.4 ± 2.2 weeks postpartum (when protein content naturally declines) whereas, in our study, preterm donor milk was expressed from delivery throughout the first four weeks postpartum.

Another strength of this study was the use of breast milk analyzer (Miris HMA) which allowed for the direct measurement of the exact protein concentration in MOM, PDM and TDM. In most studies, milk protein content is estimated using median calculated values in term donor milk [40,47] whereas in our study protein content was measured in each infant’s feeds separately. This is one of the first studies in which the protein content of pasteurized preterm donor milk is determined. Landers et al. have also mentioned the reported preterm donor human milk composition from Australian mothers in order to provide evidence for the efficacy of feeding donor human milk to premature infants [48].

Limitations of this study were associated with the difficulty in recruiting mothers delivering preterm. As a result, preterm donor milk was in limited supply and thus the donor milk period was restricted to the first three weeks of life. The limited source of preterm donor milk has also impacted the number of infants enrolled in the study.

## 5. Conclusions

This study showed that feeding VLBW infants with MOM supplemented (when necessary) with pasteurized PDM has a positive impact on protein intake, as well as on body weight and head circumference at the end of the three-week intervention. Although the absolute value of body weight was also positively impacted at discharge, Δz-scores of anthropometric characteristics studied from birth to discharge did not differ significantly between groups. Whether, in our study population, the positive impact of PDM on growth during the initial vulnerable period of life might also have beneficial effects on neurodevelopment later in life remains to be further studied. Considering the unique nutritional needs of VLBW infants, milk banks should prioritize feeding them with milk expressed from mothers delivering prematurely when MOM is unavailable or insufficient. Efforts should be made to match, if possible, the prematurity level of donor milk to that of the infant.

To our knowledge, this is one of the first studies where PDM was used and analyzed for its nutritional content. Further research is required to establish the short- and long-term effects of feeding VLBW infants with PDM if required.

## Figures and Tables

**Table 1:** Demographic and clinical parameters of the study population.

	Total Population (N = 120)	Group A (N = 43)	Group B (N = 77)	*p*-Value
	*Mean (SD)*	*Mean (SD)*	*Mean (SD)*	
Mother’s age (years)	33.8 (5.1)	32.7 (5.0)	34.4 (5.0)	0.233 +
Gestational age (weeks)	29.7 (2.5)	29.7 (2.4)	29.7 (2.5)	0.959 +
	*N (%)*	*N (%)*	*N (%)*	
Pregnancy				
Single	88 (73.3)	30 (69.8)	52 (67.5)	0.484 ‡
Multiple	32 (26.7)	13 (30.2)	25 (32.5)	
Gender				
Females	47 (39.2)	14 (32.6)	33 (42.9)	0.268 ‡
Males	73 (60.8)	29 (67.4)	44 (57.1)	
SGA	10 (8.3)	3 (7.0)	7 (9.1)	0.489 ‡
	*Mean (SD)*	*Mean (SD)*	*Mean (SD)*	
Birth Weight (g)	1169.8 (234.1)	1182.9 (257.6)	1162.5 (221.3)	0.649 +
Birth Weight z-score	−0.48 (0.98)	−0.48 (0.93)	−0.48 (1.01)	0.987 +
Body length at birth (cm)	38.0 (3.3)	38.2 (3.1)	37.8 (3.3)	0.562 +
Body length z-score	−0.20 (1.14)	−0.13 (1.21)	−0.24 (1.11)	0.598 +
Head circumference at birth (cm)	26.6 (2.0)	26.5 (2.1)	26.7 (1.9)	0.720 +
Head circumference z-score at birth	−0.24 (1.26)	−0.38 (1.25)	−0.16 (1.26)	0.362 +
CRIB II score	6.9 (3.1)	6.8 (3.1)	6.9 (3.2)	0.878 +
	*Median (IQR)*	*Median (IQR)*	*Median (IQR)*	
Length of hospitalization (days)	56.5 (45.0–69.5)	58.0 (47.0–68.0)	55.0 (42.0–72.0)	0.465 ++

Group A: VLBW infants fed with MOM supplemented, if needed, with preterm donor milk (PDM); Group B: VLBW infants fed with MOM supplemented, if needed, with term donor milk (TDM); + Student’s *t*-test; ++ Mann-Whitney test, ‡ Pearson’s chi-square test.

**Table 2:** Characteristics of donor mothers.

	Total Population (N = 69)	PDM Mothers (N = 26)	TDM Mothers (N = 43)	*p*-Value
Gestational age, weeks	34.7 (4.6)	29.8 (2.7)	38.0 (2.0)	<0.001 +
Maternal age, years	33.7 (3.7)	32.9 (4.0)	34.3 (3.4)	0.130 +
Parity, N (%)				
1st	42 (60.9)	20 (76.9)	22 (51.2)	0.138 ‡
2nd	26 (37.7)	6 (23.1)	20 (46.5)	
3rd	1 (1.4)	0 (0)	1 (2.3)	
Multiple gestation, N (%)	6 (8.7)	4 (15.4)	2 (4.6)	0.138 ‡

PDM, preterm donor milk; TDM, term donor milk; + Student’s *t*-test; ‡ Pearson’s chi-square test.

**Table 3:** Protein intake (g/kg/day) of the study population.

	Total Population (N = 120)	Group A (N = 43)	Group B (N = 77)	*p*-Value
	Mean (SD)	Mean (SD)	Mean (SD)	
During hospitalization	3.03 (0.57)	3.20 (0.60)	2.93 (0.54)	**0.014 +**
Donor milk period				
1st week	3.35 (1.05)	3.53 (1.10)	3.25 (1.01)	**0.023 ++**
2nd week	3.53 (1.13)	3.67 (1.05)	3.45 (1.17)	
3rd week	3.36 (0.99)	3.67 (0.96)	3.19 (0.97)	
At initiation of human milk fortification	3.24 (0.84)	3.57 (0.82)	2.92 (0.85)	**0.006 +**

Group A: VLBW infants fed with MOM supplemented, if needed, with preterm donor milk (PDM); Group B: VLBW infants fed with MOM supplemented, if needed, with term donor milk (TDM); in bold: significant *p*-values, *p* < 0.05; + Student’s *t*-test, ++ repeated measurements analysis of variance (ANOVA).

**Table 4:** Morbidity of the study population during hospitalization.

	Total Population (N = 120)	Group A (N = 43)	Group B (N = 77)	*p*-Value
	Ν (%)	Ν (%)	Ν (%)	
Sepsis				
No	91 (75.8)	35 (81.4)	56 (72.7)	0.288 +
Yes	29 (24.2)	8 (18.6)	21 (27.3)	
Necrotizing enterocolitis				
No	118 (98.3)	42 (97.7)	76 (98.7)	1.000 ++
Yes	2 (1.7)	1 (2.3)	1 (1.3)	
Retinopathy of prematurity				
No	116 (96.7)	41 (95.3)	75 (97.4)	0.617 ++
Yes	4 (3.3)	2 (4.7)	2 (2.6)	
Bronchopulmonary dysplasia				
No	75 (62.5)	24 (55.8)	51 (66.2)	0.258 +
Yes	45 (37.5)	19 (44.2)	26 (33.8)	
Intraventricular hemorrhage				
No	116 (96.7)	42 (97.7)	74 (96.1)	1.000 ++
Yes	4 (3.3)	1 (2.3)	3 (3.9)	
Patent Ductus Arteriosus				
No	73 (84.9)	23 (85.2)	50 (84.7)	1.000 ++
Yes	13 (15.1)	4 (14.8)	9 (15.3)	

Group A: VLBW infants fed with MOM supplemented, if needed, with preterm donor milk (PDM); Group B: VLBW infants fed with MOM supplemented, if needed, with term donor milk (TDM); + Pearson’s chi-square test; ++ Fisher’s exact test.

## Data Availability

The data presented in this study are available on request from the corresponding author. The data are not publicly available due to privacy.

## Supplementary Materials

The following supporting information can be downloaded at: https://www.mdpi.com/article/10.3390/nu15030566/s1, Figure S1: Body Weight at discharge in the two groups.

## Abbreviations

BMF, breast milk fortifier; BPD, bronchopulmonary dysplasia; CRIB, clinical risk index for babies, DM, donor milk; IVH, intraventricular hemorrhage; MOM, mother’s own milk; NEC, necrotizing enterocolitis; NICUs, neonatal intensive care units; PDA, patent ductus arteriosus; PDM, preterm donor milk; ROP, retinopathy of prematurity; SGA, small for gestational age; TDM, term donor milk; VLBW, very low birth weight.
