# Supplementary figures and images for: Supplementation of Mother’s Own Milk with Preterm Donor Human Milk: Impact on Protein Intake and Growth in Very Low Birth Weight Infants—A Randomized Controlled Study
Source: Nutrients. 2023 Jan 21;15(3):566. doi: 10.3390/nu15030566 (PMC9919101; doi:10.3390/nu15030566)

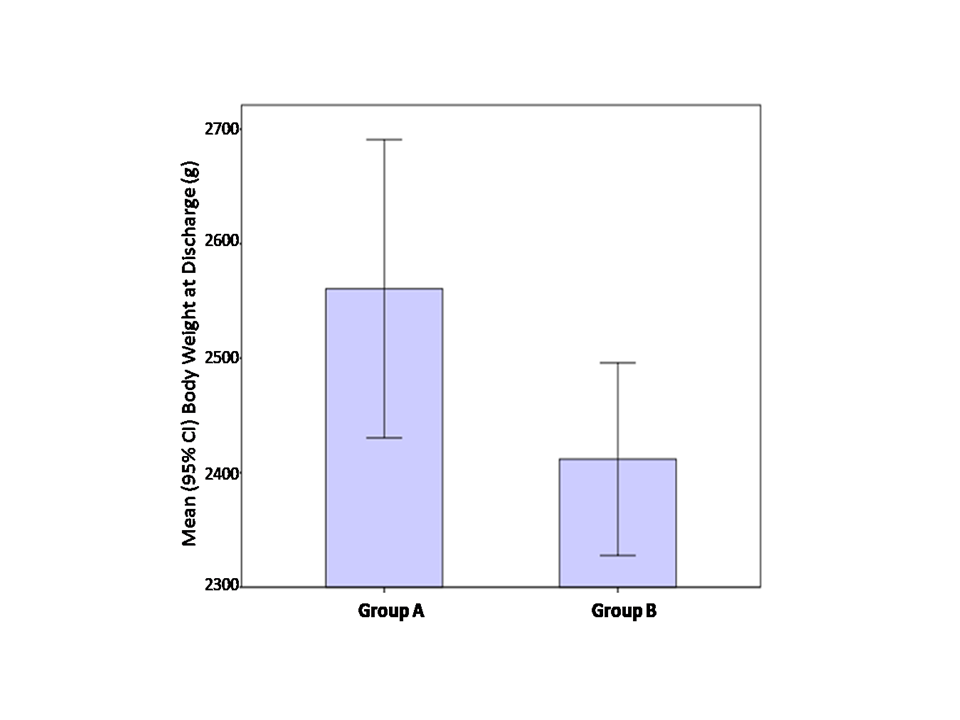

Supplement: Supplementary file 1 [file nutrients-15-00566-s001.zip › nutrients-2161328-supplementary.tif]
